# Supplementary material for: Hydrodynamical effect of parallelly swimming fish using computational fluid dynamics method
Source: PLoS One. 2021 May 3;16(5):e0250837. doi: 10.1371/journal.pone.0250837 (PMC8092777; doi:10.1371/journal.pone.0250837)
Supplement: Attachment — Submitted filename: 2021-03-30_Response_to_reviewers_Doi-et-al.docx [file pone.0250837.s001.docx]

Dear Editor and Reviewers:

Thank you very much for your helpful comments. We are enclosing a revised version of our manuscript, along with our responses to your suggestions regarding the document.

The manuscript has been carefully rechecked and appropriate changes have been made in accordance with the suggestions received. The major changes made are highlighted in yellow in the revised document. Detailed responses to all comments have also been prepared and are attached herewith.

We thank you for your thoughtful suggestions and insights, which have enriched the manuscript and produced a more balanced and better account of the research. We hope that the revised manuscript is now suitable for publication in *PLOS ONE*.

Thank you for your consideration. We look forward to hearing from you.

Yours sincerely,

Shinsuke Torisawa

**To Reviewer #1**

**Q1.** I do not agree that the major contribution is "applied the real fish kinematics to CFD analysis". Because the authors did not really apply the kinematics of real fish frame by frame. Instead, they extract the key parameters from the real fish system and then simulated by the fitted body wave function. This is done by many recent works such as:

Li, G., Kolomenskiy, D., Liu, H., Thiria, B. and Godoy-Diana, R., 2019. On the energetics and stability of a minimal fish school. PloS one, 14(8), p.e0215265.

Li, L., Nagy, M., Graving, J.M., Bak-Coleman, J., Xie, G. and Couzin, I.D., 2020. Vortex phase matching as a strategy for schooling in robots and in fish. Nature communications, 11(1), pp.1-9.

**Response**

**A1.** Thank you for your useful comments. The following text has been added to the abstract: “There are limited examples of fish models based on actual swimming movements using simulation, and the movements in these models are simple.” (Page 2 Lines 12-13)

We have also cited the previous study of Li et al. (2019 [16]) that Reviewer #1 suggested, and the following text has been added to the introduction section. “There are several examples of body wave functions applied to CFD, but their lateral excursion is simple. The body wave functions used had amplitudes such that the simple envelopes became larger as they approached the tail tip [16, 17].” (Page 6 Lines 81-82)

In addition, the following text has been added to the introduction section. “Using a pair of swimming fish robots, Li et al. [9] hypothesized that energy savings could be achieved using vortex phase-matching.” (Pages 3-4 Lines 42-44)

**Q2.** And the authors mentioned that “there are no published studies on the calculation of the thrust and drag forces from the pressure distribution on the body”; However, the truth is there are many. For example:

Dabiri, J.O., Bose, S., Gemmell, B.J., Colin, S.P. and Costello, J.H., 2014. An algorithm to estimate unsteady and quasi-steady pressure fields from velocity field measurements. Journal of Experimental Biology, 217(3), pp.331-336.

Gemmell, B.J., Colin, S.P., Costello, J.H. and Dabiri, J.O., 2015. Suction-based propulsion as a basis for efficient animal swimming. Nature communications, 6(1), pp.1-8.

Lentink, D., Haselsteiner, A.F. and Ingersoll, R., 2015. In vivo recording of aerodynamic force with an aerodynamic force platform: from drones to birds. Journal of The Royal Society Interface, 12(104), p.20141283.

I would really suggest the authors do read the related literatures and summary nicely about the contribution in the abstract and introduction.

**Response**

**A2**. Thank you for your useful comments and introducing these useful papers. The following text has been added to the introduction section. “Dabiri et al. [19] used an algorithm to determine the pressure distribution from the velocity field to obtain the pressure coefficient. Hemelrijk et al. [17] used multi-particle collision dynamics (MPCD) to estimate the thrust and drag forces acting on a fish body model. Limited work has been done to determine the thrust and drag forces acting on a fish body model using these methods.” (Pages 4-5 Lines 60-64)

However, Lentink et al. (2015) proposed a methodology of measuring thrust in a non-invasive manner, and Gemmell et al. (2015) focused on the functionality of thrust generation in aquatic animals such as jellyfish that swim mainly by suction. Therefore, we think it appropriate not to cite these two papers.

**To Reviewer #2:**

**Q1.** The strouhal number should be in the manuscript. It is not mentioned in the manuscript.

**Response**

**A1.** We highly appreciate your comment on this point. The Strouhal number of the 3D fish model has been estimated and is given in the revised manuscript. (Pages 12 Line 182-184, Page 14 Lines 231-232)

**Changes other than those the reviewers pointed out:**

The abbreviation (CFD) in the title of the paper has been changed to computational fluid dynamics. Thus, the title has been updated: "Hydrodynamical effect of parallelly swimming fish using computational fluid dynamics method"

The ethics statement was replaced Page 9 Line129-131 into Page 6 Lines 89-91 as the staff of PLOS ONE Office suggested.
